# Case of Ossification of the Ventricular Aorta

**Published:** 1821-10

**Authors:** J. Howell

**Affiliations:** Surgeon.


### *Case of Ossification of the Ventricular Aorta.* By J. Howell, Surgeon.

ROBERT PHIPPS, a strong, athletic young man, about twenty years of age, became troubled, about the beginning of last March, with a violent palpitation of the heart, attended with a slight cough and difficulty of breathing, which was considerably increased by exertion. He was blistered upon the chest, and took medicine, by which means, in a few days, he was relieved. But this relief lasted only a short time; the palpitation greatly increased, and excited in the man's mind much apprehension. He was bled a few days before I first saw him; namely, on 6th April. I found him able to be about, not exhibiting any appearance of disease, but complaining that the least exertion increased the palpitation and induced severe dyspnœa. Upon removing the clothes from his chest, the diastole of the heart could be very plainly seen, and was remarkably strong, but sometimes irregular. The pulse was about seventy, full and hard; he complained very little of his cough, and expectorated but a little mucus. In the course of twenty days I bled him largely, gave him digitalis in large doses, and frequently had recourse to purgative medicines, without producing the least effect on the action of the heart and arteries. At the end of this time he did not seem worse, if we except the debility to which the above means, and a low diet, had reduced him.

About the beginning of July, he went into St. Thomas's Hospital, and became a patient of Dr. Scott's. Here the depleting plan was pursued rigorously, and the patient confined to the milk diet; but, continuing to grow worse, he became anxious to return home, where I saw him again about the 17th July. He had been bled, he told me, fifteen times in the hospital; and, although he did not appear to have lost flesh, he was extremely weak, and unable to walk.

There seemed no diminution of the violence of the action of the heart, and the cough had become distressing, and attended with a mucous expectoration frequently mixed with blood. His lower extremities were œdematous, and he complained of violent pain in all his joints, which he continually wished his attendants to rub with their hands; and this seemed to relieve him. He was now confined to his bed, unable to lie down, frequently delirious, sleeping but for a few minutes at a time: he generally awoke with the most horrid sensations of impending suffocation. In this dreadful situation he lived a few days, and died, as if quite exhausted, on the 23d of July.

On opening the body, the lungs were found quite healthy; as indeed were all the abdominal and thoracic viscera, except the heart. There was, perhaps, about a pint of water in the chest; but, upon opening the pericardium, scarcely an ounce of fluid was found within it. Upon a very careful dissection of the heart, not a vestige of disease was found, till the left ventricle (which, as well as the left auricle, was distended with blood,) was slit open, and the beginning of the aorta brought into view; here was discovered the cause of this poor fellow's death. Just as the aorta emerges from the left ventricle, obliterating all appearance of two of the semilinear valves, there was found almost a ring of hard unequal *bone,* blocking up the vessel, so as to leave an opening a little larger than would be filled by the introduction of a large-sized quill.

A preparation has been made of this terrible malady, and certainly exhibits a rare specimen of disease.

*Wimbledon; August* 2, 1821.

---

